# Lessons from the diet: Captivity and sex shape the gut microbiota in an oviparous lizard (*Calotes versicolor*)

**DOI:** 10.1002/ece3.8586

**Published:** 2022-02-12

**Authors:** Lin Zhang, Fang Yang, Tangliang Li, Buddhi Dayananda, Longhui Lin, Chixian Lin

**Affiliations:** ^1^ School of Basic Medical Sciences Hubei University of Chinese Medicine Wuhan China; ^2^ School of Laboratory Medicine Hubei University of Chinese Medicine Wuhan China; ^3^ State Key Laboratory of Microbial Technology, Institute of Microbial Technology Shandong University Qingdao China; ^4^ School of Agriculture and Food Sciences The University of Queensland Brisbane QLD Australia; ^5^ College of Life and Environmental Sciences Hangzhou Normal University Hangzhou China; ^6^ MOE Key Laboratory of Utilization and Conservation for Tropical Marine Bioresources Hainan Key Laboratory of Herpetological Research College of Fisheries and Life Science Hainan Tropical Ocean University Sanya China

**Keywords:** *Calotes versicolor*, captivity, diet, gut microbiota, sex

## Abstract

Studies have indicated that the abundance and community structure of gut microbiota are altered by diet. In this study, next‐generation sequencing of the 16S rRNA gene amplicon was performed to evaluate variations in the gut microbiota of wild and captive individuals of both sexes of *Calotes versicolor*. The results showed that there was a significant sex difference in microbial community structure for wild *C. versicolor*, *Bacteroide* was the dominant genus in wild females (WF), whereas *Ochrobactrum* was the dominant genus in wild males (WM). *Acinetobacter* and *Hymenobacter* were the dominant genera in WF, while *Clostridium* was the dominant genus in captive females (CF). The results indicated that differences in diet between wild and captive *C. versicolor* also resulted in variations in gut microbiota. Thus, it was not surprising that captivity and sex shape the gut microbiota in *C. versicolor*. In summary, the fundamental information presented about the gut microbiota of both sexes of wild (and captive females) *C. versicolor*, indicates that the artificial environments are not suitable for the wild *C. versicolor*.

## Graphical abstract

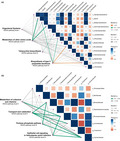

## 1 INTRODUCTION

Gut microbiota plays a critical role in host health and provides fundamental information about host physiology (Hale et al., 2018). Complex communities of gut microbiota are associated with host energy budget and nutrient metabolism (Cani, 2016; Rowland et al., 2018; Semova et al., 2012), foraging behavior (Heijtz et al., 2011), immune homeostasis (Dimitriu et al., 2013; Rastelli et al., 2019; Round & Mazmanian, 2009), and reproductive performance (Leftwich et al., 2017). Due to its size and complexity, the gut microbiota (microbiome) is known as the second genome (Weinstock, 2012). In recent years, the rapid development and decreasing cost of next‐generation sequencing has led to an increase in studies on the role of the gut microbiome in wildlife and human health (Debelius et al., 2016; Hird, 2017; Ingala et al., 2018; Zhu et al., 2011). In particular, studies have focused on explaining the coevolution of hosts and gut microbiota (Ingala et al., 2018; Montoya‐Ciriaco et al., 2020; Videvall et al., 2018), and diseases (Fan & Pedersen, 2021; Wang et al., 2021; Zhang et al., 2020).

*Bacteroidetes*, *Firmicutes*, and *Proteobacteria* are three of the most important components of the gut microbiota in vertebrate species (Kohl et al., 2017; Ren et al., 2016). Several studies have shown that gut microbiota may play a significant role in vertebrate evolution, as its diversity is correlated with the evolutionary history of these animals. However, vertebrate gut microbial communities are influenced by several other factors, such as host features (age, body size, and sex) and environment (diet and season) (Delsuc et al., 2014; Martin et al., 2010; Zhou, Nelson, et al., 2020). For example, in nonreproductive mice the relative abundance of *Lactobacillus* spp. was higher in males than in females, whereas the contrary was observed in reproductive mice (Maurice et al., 2015). Female *Sceloporus virgatus* display significantly lower microbial diversity and richness than males (Martin et al., 2010). Moreover, compared to males, *Rhinella marina* females display an increased relative abundance of *Bacteroides*, *Comamonas*, *Flavobacterium*, *Microvirgula*, *Parabacteroides*, and *Pseudomonas* species, with a decreased relative abundance of *Cetobacterium*, *Clostridium*, *Epulopiscium*, *Plesiomonas*, and *Vibrio* species (Zhou, Nelson, et al., 2020). However, no influences of sex have been observed in the microbial communities of *Liolarmus* and *Phymaturus* lizards (Kohl et al., 2017). Thus, the influence of sex on gut microbial communities in wildlife species is complex and largely unknown.

Recent studies have shown that captivity plays an important role in endangered species conservation by maintaining the breeding population, particularly for lizards; however, captivity has been shown to significantly alter the gut microbial community of lizards (Jiang et al., 2017; Kohl et al., 2017; Tang et al., 2020; Zhou, Zhao, et al., 2020), amphibians (Bataille et al., 2016; Tong et al., 2019), and other taxa (Chi et al., 2019; Hale et al., 2018; Martínez‐Mota et al., 2020; Oliveira et al., 2020). For instance, a study by Kohl et al. (2017) found that captive species exhibited less *Firmicutes* and *Actinobacteria* compared to wild species, and *Bacteroidetes* was only present in captive species. Furthermore, some studies have reported a significant difference between the alpha diversity of wild and captive animal gut microbiotas (Kohl et al., 2017; Ren et al., 2016), whereas other studies have indicated a general loss of microbial diversity as a result of captivity (Kohl & Dearing, 2014; Kohl et al., 2014).

Microbiome characterization and monitoring tools are being developed and are recommended for wild species conservation (Oliveira et al., 2020; Redford et al., 2012), particularly endangered species such as the giant panda (*Ailuropoda melanoleuca*) (Zhu et al., 2011), the Yangtze finless porpoise (*Neophocaena asiaeorientalis asiaeorientalis*) (Wan et al., 2016), and the Chinese crocodile lizard (*Shinisaurus crocodilurus*) (Jiang et al., 2017; Tang et al., 2020). Considering of the sex of individuals in captivity is important of conservation activities, particularly for health breeding programs. However, the important of gut microbiota to conservation efforts for lizards of both sexes in captive and wild environments is largely unknown.

Therefore, the main goal of this study was to characterize the gut microbiota of the lizard *Calotes versicolor* to address the following questions: (1) Do gut microbial communities vary with the sex of the lizard? (2) Does gut microbiota respond to captivity? (3) What functions are differentially determined by the bacteria? To answer these questions, we used 16S rRNA gene sequencing to characterize microbial communities sampled from: (1) wild lizards (nine individuals per sex), and (2) from lizards maintained in captivity and fed in a seminatural environment for 90 days. *Calotes versicolor* (Agamidae) is an oviparous, arboreal, multiple‐clutched, omnivorous lizard, widely distributed in Indo‐Malaya, that most feeds on *Arthropoda*, including *Diptera*, *Coleoptera*, *Lepidoptera*, and *Orthoptera* (Qiu et al., 2001). Adult lizards do not display sexually dimorphism in snout‐vent length (Qiu et al., 2001), but females have relatively narrow heads compared to males (Shanbhag & Parsad, 1993). Hindgut samples from *C. versicolor* tend to have more *Firmicutes* and *Bacteroidetes*, and less *Proteobacteria* than those of the small intestine (Zhang et al., 2021).

## 2 MATERIALS AND METHODS

### 2.1 Ethics

All experiments, including the sample collection, complied with the current laws of China for the care and use of experimental animals, were approved by Hainan Tropical Ocean University (September 2019), and followed the principles of the Ethical Committee for Experimental Animal Welfare of the Hangzhou Normal University (No. 2018135).

### 2.2 Sample collection

Eighteen healthy and nongravid adult lizards (9♀♀:9♂♂) from Hainan, China, were captured and numbered in June 2019. Hindgut samples were collected immediately after capture following Zhang et al. (2021) described, and labeled as wild females (WF) and wild males (WM). The collected lizards were maintained in captivity in a seminatural environment for 90 days, where they were fed *Tenebrio molitor* and *Gryllulus chinensis*. Hindgut samples were collected again at the end of this period. Samples from only four females were obtained after the 90‐days captivity period as most lizards had escaped, these samples were recorded as captive females (CF). All hindgut samples were collected separately and stored at −80°C prior to DNA extraction.

### 2.3 Gut microbiota analyses

DNA was extracted from all samples using the cetyltrimethylammonium bromide (CTAB)/sodium dodecyl sulfate (SDS) method. Universal primers were employed to amplify the V3–V4 regions of the bacterial 16S rRNA genes that contained Illumina sequences at the 5′‐end of forward primers harboring 7‒12 bp barcodes. Sequencing was performed using an Illumina MiSeq platform (San Diego, CA, USA) with Frasergen Bioinformatics (Wuhan, Hubei, China). All sequence analyses were performed using the QIIME software package (https://qiime.org/, Caporaso et al., 2010). Chloroplast and mitochondrial sequences were removed from the dataset. Operational taxonomic units (OTUs) with 97% similarity were defined using UCLUST (Edgar, 2010).

### 2.4 Statistical analyses

Variations in alpha diversity were analyzed using *Tukey's HSD* test. Nonmetric multidimensional scaling (NMDS) based on the Bray‐Curtis distance was constructed to determine the variations in beta‐diversity. To obtain the unique genus, features that occurred in ≥75% of the replicates in each group were retained. The linear discriminant analysis (LDA) effect size (LEfSe) method was employed to identify the variations in microbial communities based on LDA sources (Segata et al., 2011). To explore the functional profiles of gut microbiota between WM and WF, or between WF and CF, all OTUs were assigned to the Kyoto Encyclopedia of Genes and Genomes (KEGG) pathways by Tax4Fun (Aßhauer et al., 2015). The differences in gene function analyses were identified in STAMP (v2.1.3) (Parks et al., 2014), and *Welch's t*‐test was used for the comparisons between the two groups.

The relationships between various microbial communities were analyzed using Spearman's correlation coefficients. Partial Mantel tests were performed to evaluate relationships between the relative abundance of microbiota and gene functions. All analyses were conducted using the *linKET* package (Huang, 2021) in R version 4.0.4 (R Core Team, 2021).

## 3 RESULTS

**FIGURE 1:**
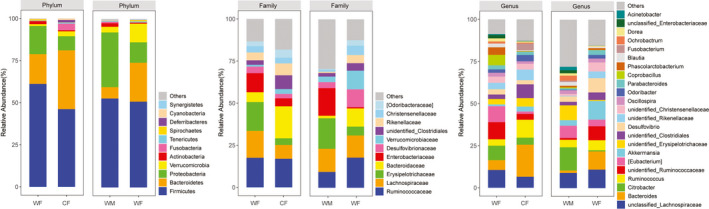
Composition of the gut microbiota of each group at the phylum, family and genus levels

In all lizards, *Firmicutes*, *Proteobacteria*, *Bacteroidetes*, and *Verrucomicrobia*, were the four dominant phyla identified (mean relative abundance >1%, Figure 1a). *Actinobacteria* only was a dominant phylum in WM, but not in WF (Figure 1a). Richness, Chao1, and ACE indices were lower in WF than in WM (all *adj p* < .01), but Shannon, Simpson, and Pielou's E diversity indices showed no differences (all *adj p* > .05) between WM and WF (Figure S1).

*Fusobacteria* and *Deferribacteres* were the two dominant phyla in CF, but *Actinobacteria* was a dominant phylum in WF. Captivity was correlated with the loss of the *Firmicutes* and *Proteobacteria*, and an introduction of the *Bacteroidetes* and *Verrucomicrobia* (Figure 1a). Nevertheless, the Shannon, Richness, Simpson, Pielou's E, Chao1, and ACE indices showed no differences (all *adj p* > .05) between WF and CF (Figure S2).

**FIGURE 2:**
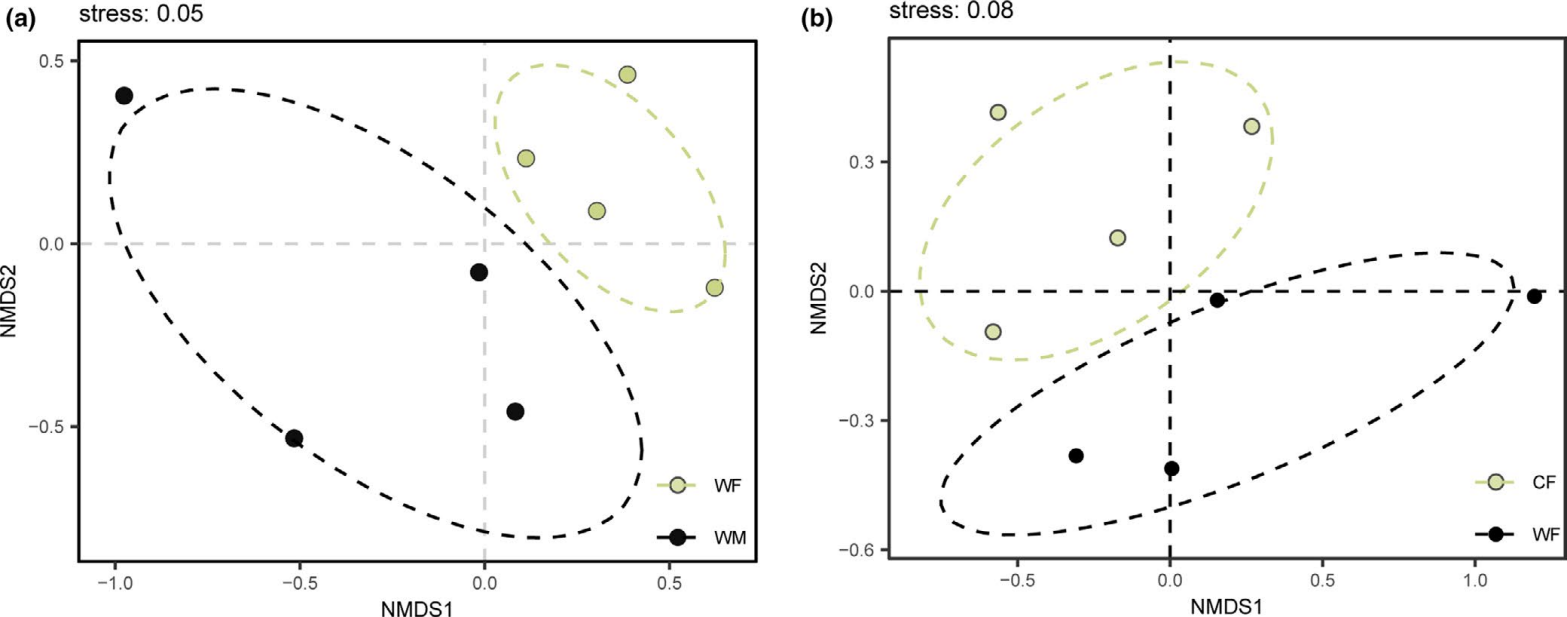
The non‐metric multidimensional scaling (NMDS) of the gut microbiota composition. The variation explanation is indicated on each axis, respectively

The NMDS plots showed significant differences in beta diversity between WF and WM (Figure 2a), and WF and CF (Figure 2b), respectively (Adonis test: WM‐WF, *R* ^2^ = .204, *p* = .03; WF‐CF, *R* ^2^ = .230, *p* < .01).

**FIGURE 3:**
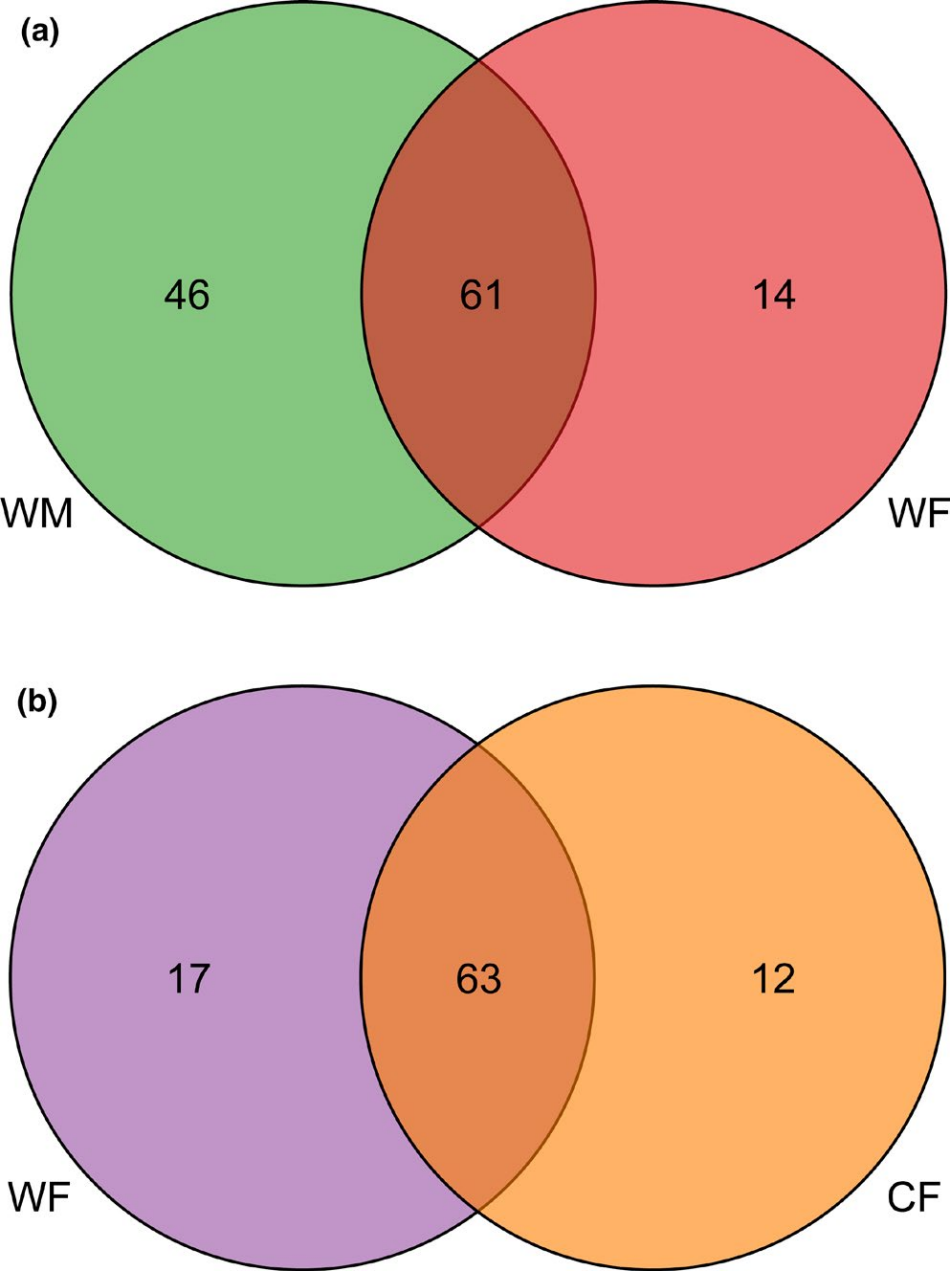
The Venn plot to show the unique and share genus between WM and WF (a) and WF and CF (b)

We obtained 75, 107, 80, and 75 genera from WF and WM as well as WF and CF, respectively. WM and WF shared 61 genera (Figure 3a), but 46 and 14 genera were unique to WM and WF, respectively. In addition, WF and CF shared 63 genera (Figure 3b), but 17 and 12 genera were unique to WF and CF, respectively.

**FIGURE 4:**
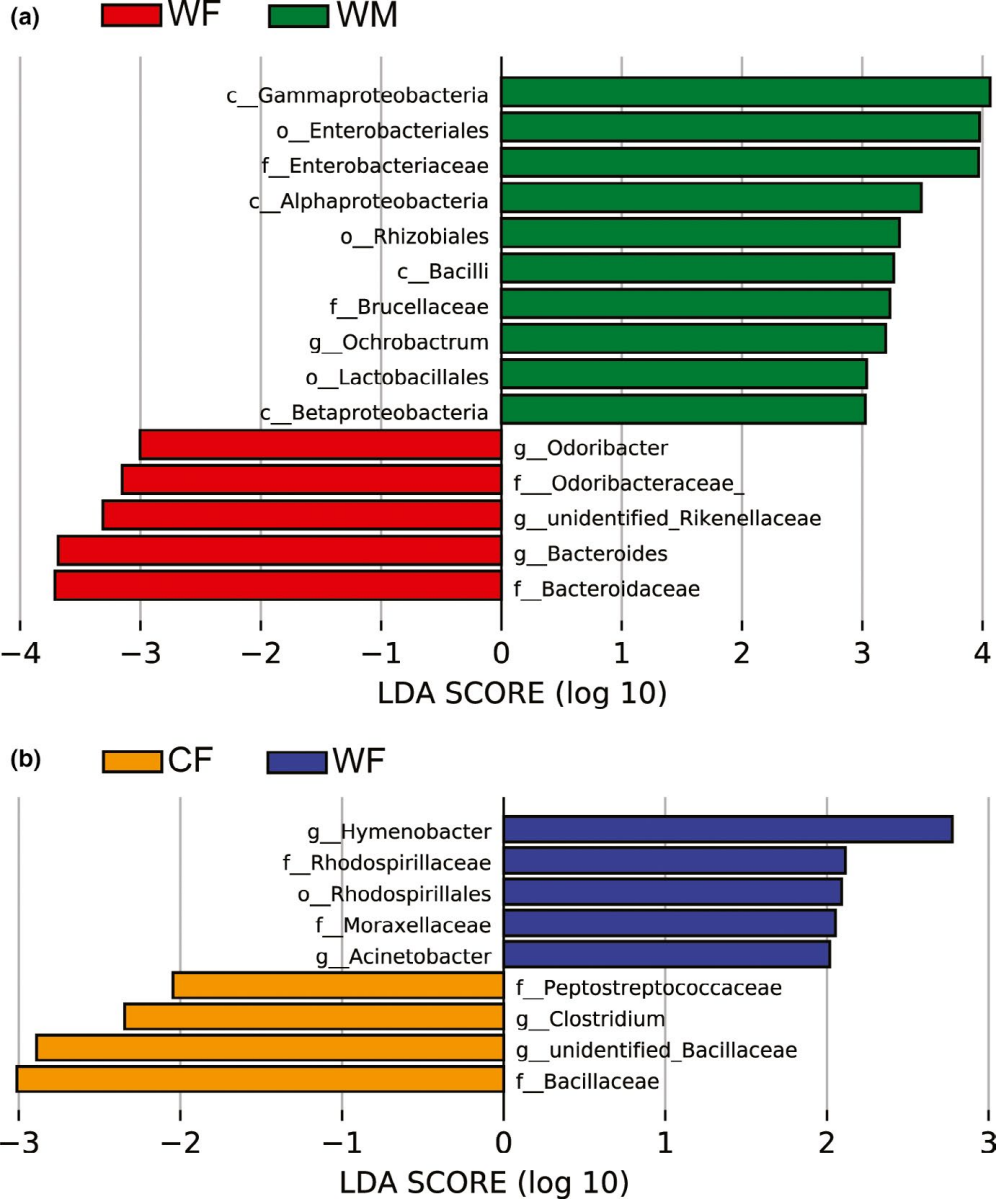
Linear discriminative analysis of effect size (LEfSe) analysis of taxonomic biomarkers of gut microbiota. (a) Cladogram of significant changes at all taxonomic levels. The root of the cladogram represents the domain bacteria. The size of node represents the abundance of taxa. (b) Histogram of the LDA score computed for features differentially abundant taxon. LDA score >4 were shown

Linear discriminant analysis effect size was performed to identify the specific bacterial taxa in samples and to compare the gut microbiota of WF and WM (LDA > 3.0, *p* < .05, Figure 4a), and WF and CF (LDA > 2.0, *p* < .05, Figure 4b). In the first comparison, four discriminative features at the class level (*Alphaproteobacteria*, *Bacilli*, *Betaproteobacteria*, and *Gammaproteobacteria*), and three at the order level (*Enterobacteriales*, *Lactobacillales*, and *Rhizobiales*) were identified in the WM; at the family level, two discriminative features (*Brucellaceae* and *Enterobacteriaceae*) were identified in WM, and two discriminative features (*Bacteroidaceae* and *Odoriobacteraceae*) in WF, and at the genus level, one discriminative feature (*Ochrobactrum*) were identified in WM, and two discriminative features (*Bacteroides* and *Odoribacter*) in WF.

From the comparison made after the captivity period, at order level, one discriminative feature (*Rhodospirillales*) was found in the CF; at family level, two discriminative features (*Moraxellaceae* and *Rhodospirillaceae*) were identified in the CF, and two discriminative features (*Bacillaceae* and *Peptostreptococcaceae*) in the WF; and at genus level, two discriminative features (*Acinetobacter* and *Hymenobacter*) were identified in CF, and one discriminative feature (*Clostridium*) in WF.

**FIGURE 5:**
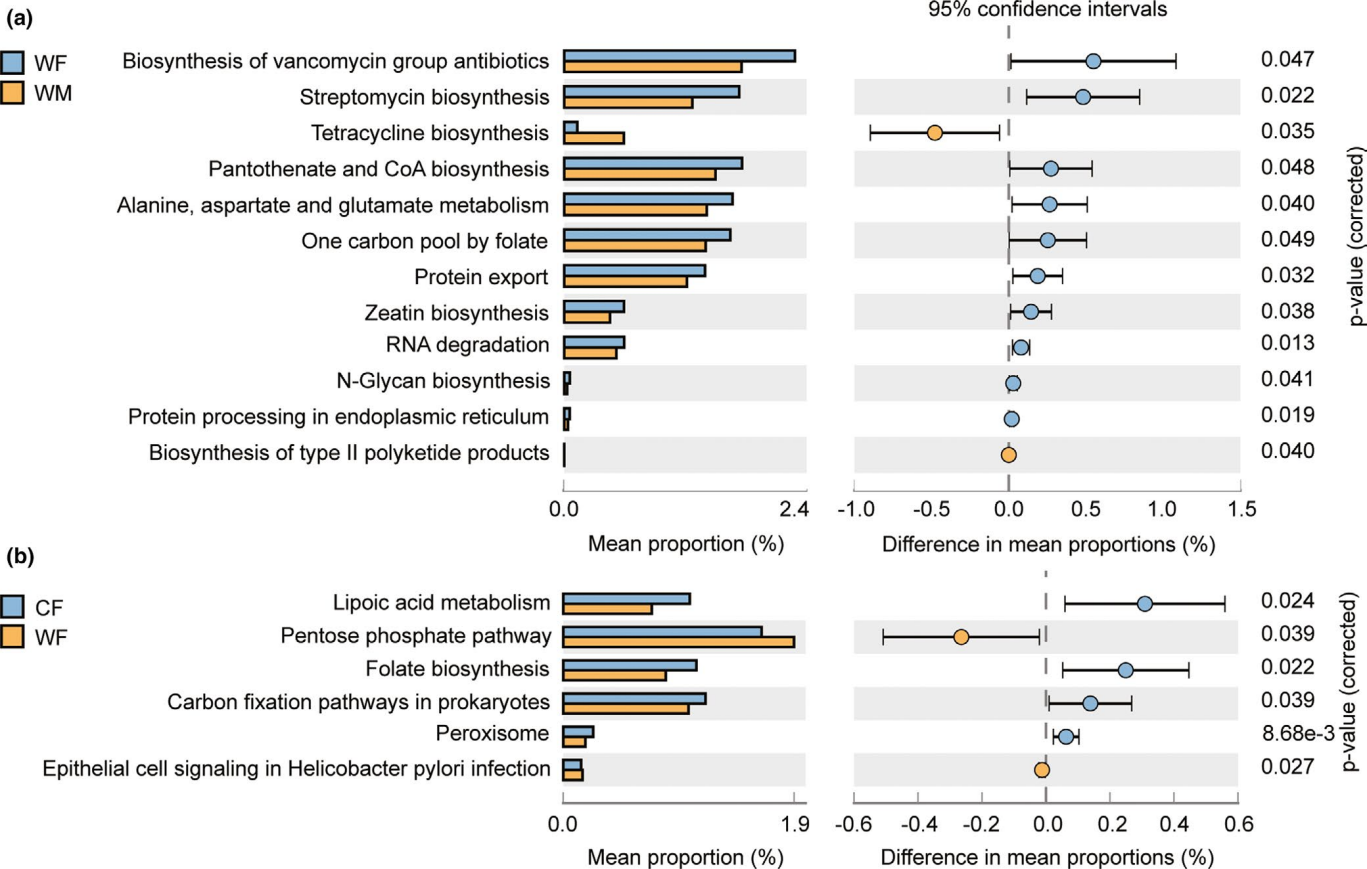
Functionally predicted KEGG pathways differing in (a) between wild males and wild females and (b) between wild females and captive females of *Calotes versicolor*. The bar plot shows mean proportions of differential level 3 of KEGG pathways predicted using Tax4Fun. The difference in proportions between the groups is shown with 95% confidence intervals. Only *p* value < .05 (Welch's *t*‐test, FDR adjusted) are shown and composition

There were including 172 (for sex) and 169 (for wild *vs* captivity) KEGG metabolic pathways were selected between WF and WM, and WF and CF, respectively, associated with metabolism (68.60% and 68.05%), genetic information processing (10.47% and 10.65%), cellular processes (3.49% and 5.33%), environmental information processing (4.07% and 3.55%), organismal systems (2.91% and 3.01%), and human diseases (9.30% and 8.28%). There was significant difference in organismal systems between WF and WM (adj *p* = .038), but no significant differences were detected between WF and CF (adj *p* > .20). Functional predictions identified 12 differentially present level 3 KEGG pathways between WF and WM (Figure 5a). The tetracycline biosynthesis and biosynthesis of type II polyketide product pathways were higher in WM, whereas other pathways were higher in WF. Six pathways differed between WF and CF (Figure 5b). The pentose phosphate pathway and epithelial cell signaling of the *Helicobacter pylori* infection were higher in WF, while other pathways were higher in CF.

**FIGURE 6:**
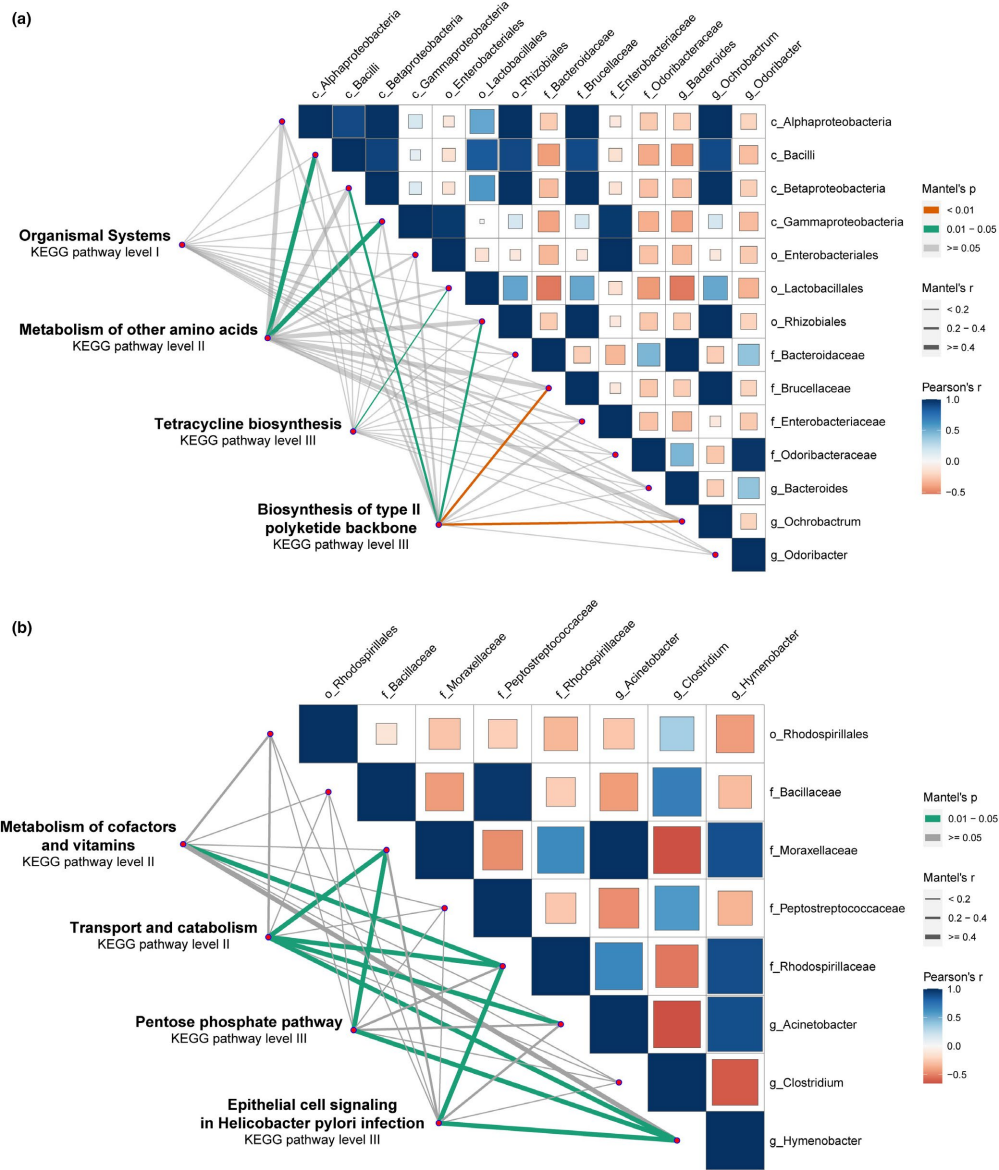
Relationships between bacterial and KEGG pathway by sex (a) and captive (b). Pairwise comparisons of bacterial were displayed with a color gradient denoting Spearman's correlation coefficient. Bacterial and KEGG community composition was related to each bacterium by Mantel test

Some discriminative features in WF and WM (Figure 6a) or WF and CF (Figure 6b) were negatively correlated. In the WM group, *Bacilli* and *Gammaproteobacteria* had a significant effect on the metabolism of other amino acids; *Lactobacillales* had a weak effect on tetracycline biosynthesis; and *Betaproteobacteria*, *Rhizobiales*, *Brucellaceae*, and *Ochrobactrum* had a significant effect on biosynthesis of the type II polyketide backbone. In the CF group, *Moraxellaceae*, *Rhodospirillaceae*, *Acinetobacter*, and *Hymenobacter* had a significant effect on the metabolism of cofactors and vitamins, transport and catabolism, pentose phosphate pathway, and epithelial cell signaling in the *H. pylori* infection.

## 4 DISCUSSION

Our results revealed that the core microbiota of *C. versicolor* consisted of *Firmicutes*, *Proteobacteria*, *Bacteroidetes*, and *Verrucomicrobia* at the phylum level, which is consistent with the results of previous studies (Jiang et al., 2017; Kohl et al., 2017; Tang et al., 2020; Zhou, Zhao, et al., 2020). Furthermore, our results showed that sex does influence gut microbial communities, with WF having significantly lower gut microbial diversity and richness compared to WM. Previous studies have detected sex‐related differences in the gut microbiota of *S*. *virgatus* (Martin et al., 2010), and *R*. *marina* (Zhou, Nelson, et al., 2020). Sexual dimorphism may be related to differences in the spatial and temporal niches (Butler, 2007), with male lizards having higher a perch than females for defending territories and remaining visible to potential mates (Logan et al., 2021). However, the snout‐vent length of *C. versicolor* is not sexually dimorphism (Qiu et al., 2001). A previous study showed that sex hormones intercept changes in gut microbiota via gonadectomy and testosterone hormone replacement in mice (Org et al., 2016). Hormonal changes and sex differences strongly affect bile acid profiles (Org et al., 2016), which respond to high‐fat/high‐sugar diets, which in turn affect gut microbiota (Islam et al., 2011; Li & Chiang, 2015). Moreover, the relatively narrow heads of female *C. versicolor* may be related to the selection of a small‐sized diet. The food niche overlap between sexes is 0.522 (Qiu et al., 2001). The main diet of adult females includes Orthoptera (Acrididae, 9.0%), Coleoptera (Chrysomelidae 14.7% and Scarabaeoidae 9.0%), and Diptera (Platypezidae 34.6%), whereas that of adult males includes Orthoptera (Acrididae 11.6%), Coleoptera (Chrysomelidae 24.5%), and Lepidoptera (Nymphalidae 11.1% and Papilionidae 17.6%) (Qiu et al., 2001). In the present study, we found that *Bacteroides* was the most dominant genus in WF, with *Ochrobactrum* being the dominant genus in WM. *Bacteroides* can employ dietary or host‐derived glycans and proteins according to the nutrient availability (Sonnenburg et al., 2005), improving the utilization rate of assimilated nutrients and incorporating external amino acids. The LEfSe showed that *Bacilli*, *Gammaproteobacteria*, *Lactobacillales*, *Betaproteobacteria*, *Rhizobiales*, *Brucellaceae*, and *Ochrobactrum* were discriminative features in the WM group. Based on the relationships between bacterial and KEGG pathways, we found that *Bacilli* and *Gammaproteobacteria* had a significant effect on the metabolism of other amino acids, *Lactobacillales* had a weak effect on tetracycline biosynthesis, and *Betaproteobacteria*, *Rhizobiales*, *Brucellaceae*, and *Ochrobactrum* had a significant effect on the biosynthesis of the type II polyketide backbone. Thus, the differences in diets may have directly driven variations in the gut microbial communities of *C. versicolor* males and females.

Previous studies have shown that captivity influences gut microbiota (Tang et al., 2020; Zhou, Zhao, et al., 2020). Our results showed that captivity was related to a loss of *Firmicutes* and *Proteobacteria*, and the introduction of *Bacteroidetes* and *Verrucomicrobia* to gut microbiota. *Firmicutes* play an important role in fiber and cellulose degradation by breaking down cellulose into volatile fatty acids, which can be used by the host. The higher abundance of *Firmicutes* in wild lizards probably leads to improved digestion and absorption of nutrients. Captive lizards were fed with an artificial fodder composed of *Tenebrio molitor* and *Gryllulus chinensis*, which had a relatively high‐fat content, but a relatively low fiber content. Moreover, two discriminative features (*Acinetobacter* and *Hymenobacter*) were identified in CF, while another (*Clostridium*) was identified in WF. *Clostridium* was positively correlated with the serum levels of total cholesterol, low‐density lipoprotein cholesterol, and triacylglycerols (Guo et al., 2018). The LEfSe showed that *Moraxellaceae*, *Rhodospirillaceae*, *Acinetobacter*, and *Hymenobacter* were discriminative features in the WF group. The relationships between bacterial and KEGG pathways indicated that the presence of *Moraxellaceae*, *Rhodospirillaceae*, *Acinetobacter*, and *Hymenobacter* had a significant effect on the metabolism of cofactors and vitamins, transport and catabolism, pentose phosphate pathway, and epithelial cell signaling in the *H. pylori* infection. Therefore, the results of this study indicated that a simple diet in captivity directly influences the gut microbial communities of *C. versicolor*.

## 5 CONCLUSIONS

In conclusion, the bacterial phyla *Firmicutes*, *Proteobacteria*, *Bacteroidetes*, and *Verrucomicrobia* dominated the core microbiota of *C. versicolor*. Our results led to the following conclusions: (1) WF having significantly lower microbial diversity and richness compared to WM; (2) captivity is related to a loss of *Firmicutes* and *Proteobacteria*, but also to the introduction of *Bacteroidetes* and *Verrucomicrobia*; (3) metabolic functions were differentially determined by the bacterial variations. The types and size of food items in the diet were significantly different for WM and WF, as well as CF and WF. It was not surprising we found that captivity and sex influence the gut microbiota in *C. versicolor*. The relationship between bacterial and KEGG pathways indicated that the artificial environments used here are not suitable for wild *C. versicolor*.

## CONFLICT OF INTEREST

The authors declare no conflict of interest.

## AUTHOR CONTRIBUTIONS

**Lin Zhang:** Conceptualization (equal); Data curation (equal); Formal analysis (equal); Funding acquisition (equal); Methodology (equal); Project administration (equal); Supervision (equal); Visualization (equal); Writing – original draft (equal); Writing – review & editing (equal). **Fang Yang:** Data curation (equal); Formal analysis (equal); Writing – original draft (equal). **Tangliang Li:** Data curation (equal). **Buddhi Dayananda:** Writing – review & editing (equal). **Longhui Lin:** Resources (equal). **Chixian Lin:** Funding acquisition (equal).

## Supporting information

Supplementary Material

## Data Availability

Sequencing data were exported as individual fastq files and have been deposited in Sequence Read Archive (SRA) NCBI (https://www.ncbi.nlm.nih.gov/) under the accession code: PRJNA673584 (https://www.ncbi.nlm.nih.gov/sra/PRJNA673584).
