# Supplementary material for: Lessons from the diet: Captivity and sex shape the gut microbiota in an oviparous lizard (Calotes versicolor)
Source: Ecol Evol. 2022 Feb 12;12(2):e8586. doi: 10.1002/ece3.8586 (PMC8840884; doi:10.1002/ece3.8586)

**Figure S1** The alpha diversity of the gut microbial composition from the WF and WM.

**
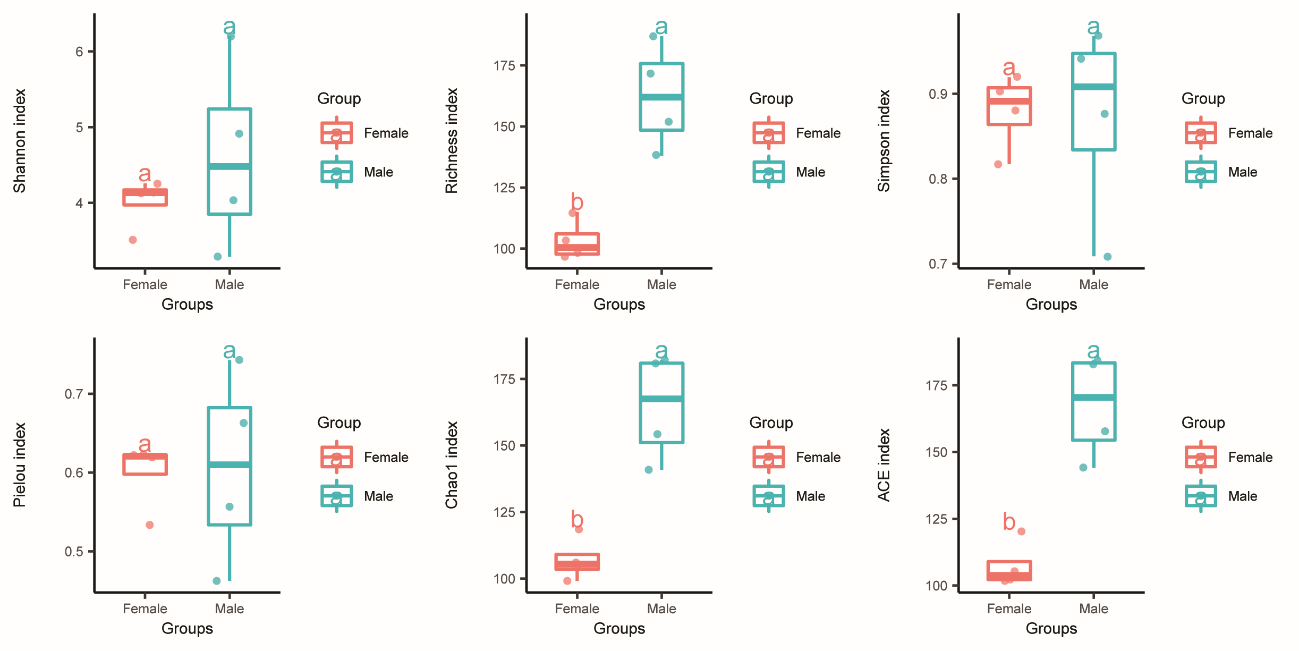
**

**Figure S2** The alpha diversity of the gut microbial composition from the WF and GF.

**
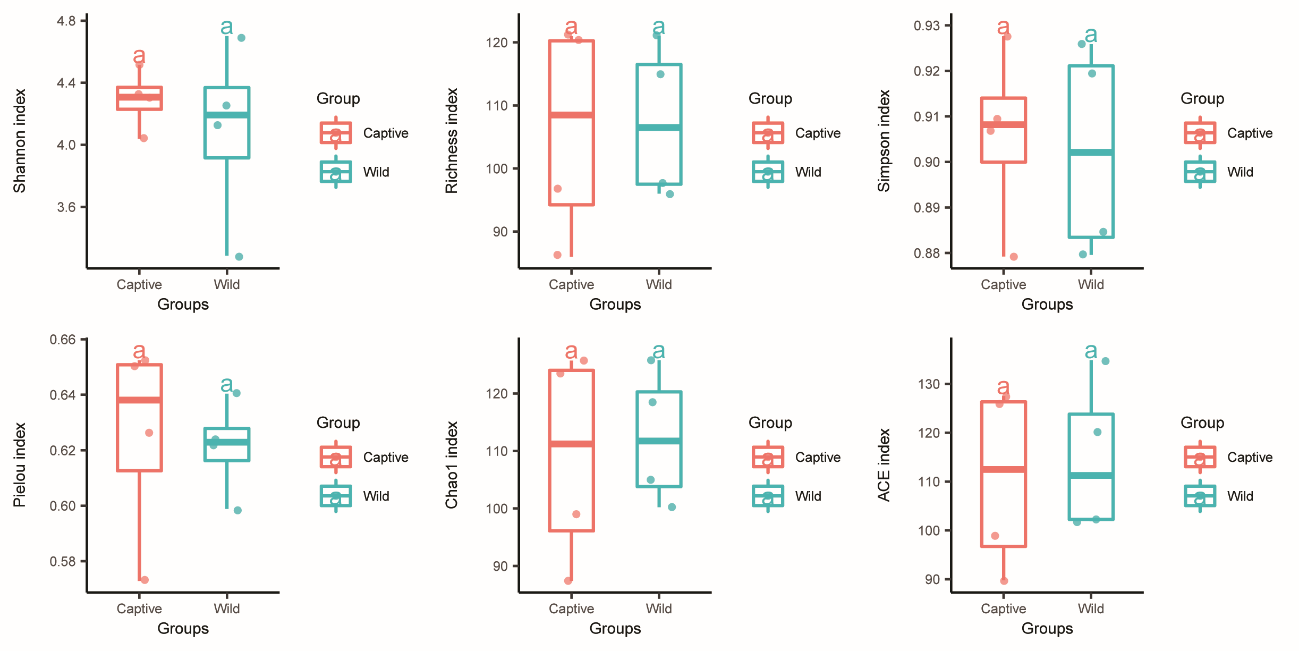
**

**Figure S3** Alpha diversity index curve. Shannon index curve (A, C) and Pielou index curve (B, D) for Sex and captivity, respectively.

**
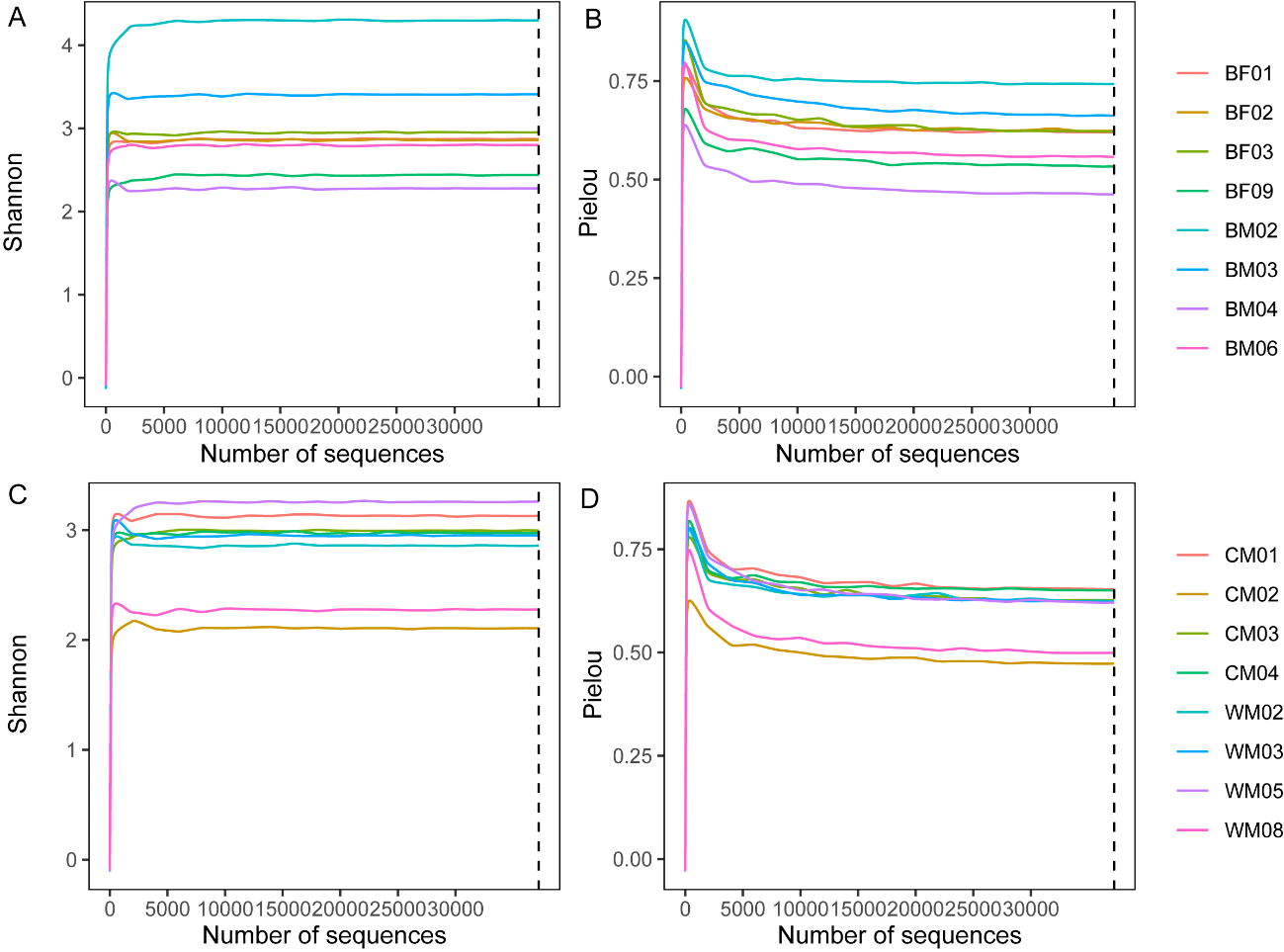
**

**Figure S4** The PCoA of the gut microbiota composition of A WF vs WM and B CF vs WF. The variation explanation is indicated on each axis, respectively.


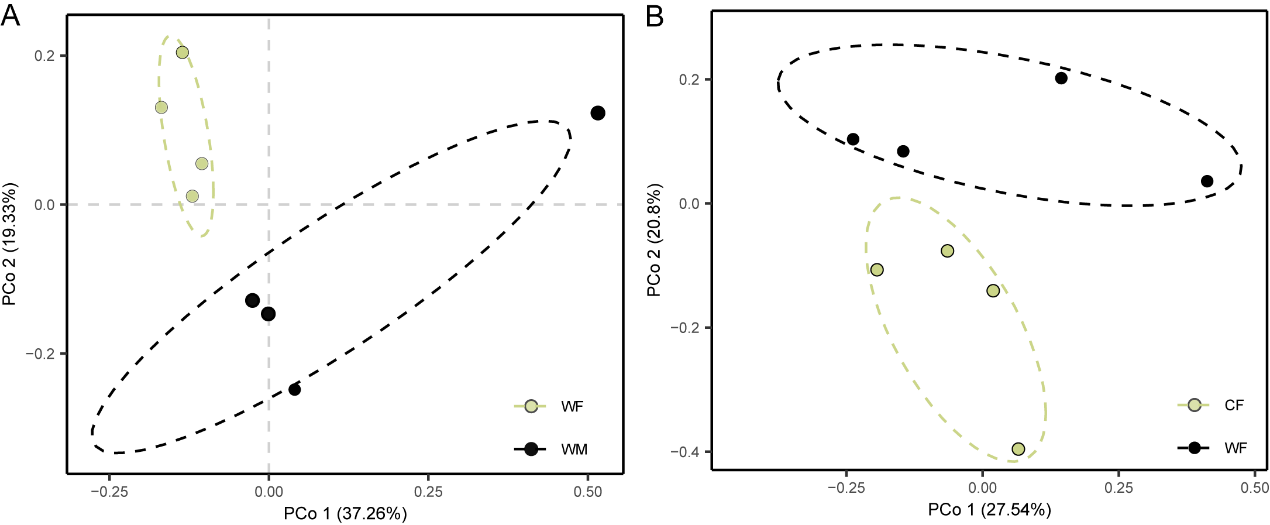

Supplement: Supplementary file 1 — Supplementary Material [file ECE3-12-e8586-s001.docx]
